# Identifying putative breast cancer-associated long intergenic non-coding RNA loci by high density SNP array analysis

**DOI:** 10.3389/fgene.2012.00299

**Published:** 2012-12-21

**Authors:** Zhengyu Jiang, Yan Zhou, Karthik Devarajan, Carolyn M. Slater, Mary B. Daly, Xiaowei Chen

**Affiliations:** ^1^Cancer Biology Program, Fox Chase Cancer CenterPhiladelphia, PA, USA; ^2^Department of Biostatistics and Bioinformatics, Fox Chase Cancer CenterPhiladelphia, PA, USA; ^3^Department of Clinical Genetics, Fox Chase Cancer CenterPhiladelphia, PA, USA

**Keywords:** long intergenic non-coding RNA (lincRNA), copy number variation (CNV), SNP array, breast cancer

## Abstract

Recent high-throughput transcript discoveries have yielded a growing recognition of long intergenic non-coding RNAs (lincRNAs), a class of arbitrarily defined transcripts (>200 nt) that are primarily produced from the intergenic space. lincRNAs have been increasingly acknowledged for their expressional dynamics and likely functional associations with cancers. However, differential gene dosage of lincRNA genes between cancer genomes is less studied. By using the high-density Human Omni5-Quad BeadChips (Illumina), we investigated genomic copy number aberrations in a set of seven tumor-normal paired primary human mammary epithelial cells (HMECs) established from patients with invasive ductal carcinoma. This Beadchip platform includes a total of 2,435,915 SNP loci dispersed at an average interval of ~700 nt throughout the intergenic region of the human genome. We mapped annotated or putative lincRNA genes to a subset of 332,539 SNP loci, which were included in our analysis for lincRNA-associated copy number variations (CNV). We have identified 122 lincRNAs, which were affected by somatic CNV with overlapped aberrations ranging from 0.14% to 100% in length. lincRNA-associated aberrations were detected predominantly with copy number losses and preferential clustering to the ends of chromosomes. Interestingly, lincRNA genes appear to be less susceptible to CNV in comparison to both protein-coding and intergenic regions (CNV affected segments in percentage: 1.8%, 37.5%, and 60.6%, respectively). In summary, our study established a novel approach utilizing high-resolution SNP array to identify lincRNA candidates, which could functionally link to tumorigenesis, and provide new strategies for the diagnosis and treatment of breast cancer.

## Introduction

Long intergenic non-coding RNAs (lincRNAs) are intergenic region-derived, large transcripts (>200 nucleotides) that do not give rise to proteins. lincRNAs, together with other long non-coding RNAs (lncRNAs), such as antisense, interleaved, and protein-coding-gene-overlapped lncRNAs, are actively transcribed from the genome, and consist of a substantial fraction of the human transcriptome (Carninci et al., 2005; Mattick and Makunin, 2006). Approximately 20–40% of the total genome is estimated to produce lncRNAs vs. less than 2% represented by protein-coding mRNAs (Nagano and Fraser, 2011), which stimulated intensive interest in the exploration of the gene structures, expressional signatures and functionality of lncRNAs. Long intergenic non-coding RNAs (lincRNA) have recently come to our attention as a result of an increased intergenic coverage of whole transcriptome and sequencing analysis.

By means of various high throughput approaches, the population of identified human lincRNA transcripts is rapidly expanding—from a class of ~3300 identified using chromatin-state mapping (Khalil et al., 2009) to a substantial catalog of over 8000 assembled from four billion RNA-seq reads (Cabili et al., 2011). This number, though reaching as much as one-fifth of mRNAs, represents a conservatively lower estimate of lincRNAs, most of which remain unannotated. Moreover, the nucleotide sequences of lincRNAs appear to be evolutionarily conserved among mammals, especially their promoters that exhibit a strong conservation resembling known protein-coding genes, suggesting they are functional despite the lack of protein-coding capacity (Guttman et al., 2009, 2010). Indeed, accumulating evidence has demonstrated various functionalities of annotated lincRNAs or even specific individual lincRNAs in mammals and humans (Gupta et al., 2010; Huarte et al., 2010; Keniry et al., 2012; Yoon et al., 2012). In addition to their tissue- and cell-specific expression signatures (Cabili et al., 2011), epigenetic regulation of gene expression, and involvement in developmental processes (Loewer et al., 2010; Guttman et al., 2011), dysregulation in cancers and their functional mechanisms involved in tumor development, including breast tumorigenesis, have begun to be investigated. Notably, a recent finding showed that numerous lincRNAs in the HOX loci demonstrated differential expression in primary breast carcinomas and metastases, particularly with HOTARI expression being up-regulated by thousands of fold, leading to the proposal that it is an independent prognostic biomarker for breast cancer metastasis (Gupta et al., 2010).

Breast cancer is among the most lethal malignant diseases in women (Howlader et al., 2012). Analyses of this disease using existing large-scale whole-genome technologies have revealed complex genomic aberrations which are believed to be the driving force of its initiation or progression (Stephens et al., 2009; Banerji et al., 2012; Ha et al., 2012). In addition to point genomic changes, large-scale structural alterations, including genomic copy number variation (CNV) and loss-of-heterozygosity (LOH) in breast tumors, though less well-understood, appear to be major contri-butors to this tumorigenesis.

CNV typically refers to genomic changes in terms of variable numbers of copies of a >1 kb DNA segment in comparison to a reference genome (Feuk et al., 2006). CNV regions of the genome are often mapped by many dosage-sensitive genes since copy gains or losses of these genes primarily determine their expression (Feuk et al., 2006; Zhou et al., 2011). Studies on correlations between CNVs of protein coding genes and corresponding expression levels, have indicated that approximately 60% of cellular expression variation and expression-associated phenotypes are accounted for by CNVs (with the exception of brain) (Henrichsen et al., 2009). Recent SNP array data from a collection of over 2000 breast cancer samples indicated that inherited and somatically acquired tumor CNVs had a strong influence on the expression of approximately 40% of genes (Curtis et al., 2012). While the majority of these array analyses have largely emphasized the protein-coding genes, none of the reports have included investigation of the intergenic non-coding genes harboring CNVs. This is likely due to the lack of high-density array coverage and inadequate annotations in intergenic regions.

Since genetic structural variations of breast cancer-associated lincRNAs have not been reported, we sought out to examine the CNV of lincRNA genes between tumors and matched adjacent normal host counterparts using HumanOmni5 Quad Beadchips. This platform provides a high coverage of the intergenic portion of the genome for high-resolution aberration detection. By using this technology, we hope to identify lincRNA associated genomic variations that may influence the progression of this disease.

## Materials and methods

### Paired biopsies and primary epithelial culture

The breast tumors and adjacent non-tumor tissues were obtained from breast cancer patients who underwent lumpectomy/mastectomy from 2008 to 2009, with informed consent as approved by the Institution Review Board (IRB) at the Fox Chase Cancer Center (FCCC). The pathological histotypes of tumors included in this study were all invasive ductal carcinoma (IDC) at stage II or greater (Table 1). Tumor cells and matched normal epithelial cells were enriched and cultured according to a commercial Human Mammary Epithelial Cell Culture Protocol (Stemcell technologies, Vancouver, BC, Canada), and maintained the same procedure for all the samples. Briefly, breast tissues were incubated and minced in EpiCult®-B Medium supplemented with 5% fetal bovine serum. Tissue fragments were then suspended in an addition of 1 × Collagenase/Hyaluronidase solution to the same medium and rotated overnight at 37°C. Contaminated fat was removed from the overlying liquefied layer following a centrifugation at 80× g for 30 s. Epithelial organoid enriched pellets were resuspended in Trypsin-EDTA (0.25%) for 1–3 min and washed in Hanks' Balanced Salt Modified Solution supplemented with 2% FBS. Occasional cell clumps were briefly dissociated in 2 mL of 5 mg/mL Dispase and 200 μL of 1 mg/mL DNase I. Single epithelial suspensions were further filtered through a 40 μm cell strainer, pelleted and cultured.

**Table 1:** **Clinical information for breast cancer patients**.

**Case ID**	**Age/Sex**	**Tumor type**	**Tumor size^a^, cm**	**IHC marker status**			**Grade^b^**
				**ER**	**PR**	**HER2-Neu**	
BC-1	49/F	IDC^c^	3.3	−	−	−	III
BC-2	40/F	IDC	5.5	−	−	+	III
BC-3	50/F	IDC	0.8	−	−	+	III
BC-4	56/F	IDC	0.2	−	−	+	II
BC-5	38/F	IDC	3.2	+	+	−	II
BC-6	62/F	IDC	5.5	+	+	E^d^	II
BC-7	33/F	IDC	1.8	+	+	−	III

a Tumor size is determined at greatest dimension.

b Grading is according to modified Bloom-Richardson grade.

c IDC, Invasive Ductal Carcinoma.

d Equivocal: negative by FISH.

### Genomic DNA isolation and SNP array

Genomic DNAs used in the global CNV analysis here were derived from paired normal-tumor primary human mammary epithelial cell lines (passage < 6) as previously described (Godwin et al., 1994). These primary HMEC lines represent the native breast normal and malignant epithelial cell populations, which we believe provides a unique advantage to studying breast cancer-associated lincRNA. The microarray assay was performed using the Infinium HD Human Omni-5 Quad Beadchip (Illumina Inc., San Diego, CA, USA). This SNP array consists of >4.3 million SNPs selected from the International HapMap Project and the 1000 Genome Project. A high density coverage of intergenic regions of the genome with over 2.4 million markers (average mean marker spacing of ~700 nt bp) provides an ideal tool to analyze lincRNA genes. For the SNP array, 400 ng (nanograms) of DNA, quantified by PicoGreen assay, were subjected to whole genome application and fragmentation, and applied to HumanOmni5 beadarrays. The scanned signal raw intensities for each array were assessed and analyzed with GenomeStudio Software (Illumina Inc., San Diego, CA, USA) using default normalization to generate X and Y intensity values for A and B alleles (generic labels for two alternative SNP alleles), respectively. An Illumina cluster file for HumanOmni5, generated by using a set of more than 200 HapMap samples, was used to computate two types of measurements for each probe: log *R* ratios (LRRs, log_2_ *R*_subject_/*R*_expected_) and B allele frequencies [BAFs, B/(A + B)], which provide information on copy number and genotyping.

### CNV analysis using Nexus

Tab-delimited bead summary data containing LRRs and BAFs were exported using a Nexus plug-in in the GenomeStudio, and CNV detection was performed using Nexus 6.0 (BioDiscovery, CA). Samples were loaded into Nexus by using a sample descriptor with matched pair design, where data from the normal sample are subtracted from that of the tumor sample to produce one result. Data from two paired replicate arrays were combined for normal samples and tumors, respectively. Probes mapping to the Y chromosome and SNPs with incorrect genotyping were filtered prior to importation to Nexus. Samples are subjected to quality assessment with a quality score below 0.02 (where the lower value represents a better quality). A linear correction was used to correct GC wave, and SNP-FASST2 segmentation algorithm was applied to generate CNV calls. A customized lincRNA annotation track was created on the basis of reference lincRNA (described below) to aid lincRNA specific CNV detection. Since the Nexus default gene track includes genes for known protein-coding as well as some of the non-coding genes, a customized protein-coding gene specific annotation track was also created to analyze CNV for the protein coding genes, which serves as an internal control for lincRNAs.

### lincRNA loci database

lincRNA reference is retrieved from the online genomic database Ensembl release 67, built on the Genome Reference Consortium release GRCh37 using the BioMart data management system. At Ensembl, lincRNA genes are *in silico* annotated using cDNA sequences (1) mapping to chromatin methylation sites outside protein coding loci, and (2) lacking protein-coding potentials (Ensembl). Given the dynamics of the number of lincRNAs in the database, other possibly relevant non-coding transcripts were also included to create a putative list. This list consisted of 6459 lincRNA and non-coding genes with starting and ending coordinates. Next, the intergenic SNPs from HumanOmni5 were subsetted and used to query the lincRNA putative list to remove any transcripts that were not present in intergenic regions to obtain a full probe list of 332,540 and a final reference list of 5800 lincRNA genes (**Table S1**). A protein-coding reference consisting of 22,088 genes was also obtained from Ensembl for the generation of a customized track in Nexus. Frequencies of CNV calls for lincRNA or genic regions from seven matched tumor-normal pairs were retrieved using the aggregate function of the Nexus package. Data were then formatted and input into Circos (http://circos.ca/, Krzywinski et al., 2009) using Perl (http://www.cpan.org/ports). Whole genome views of CNV frequencies for lincRNA or genic regions were created in PNG format.

### Quantitative real-time PCR (qPCR)

Quantitative PCR (qPCR) was performed in the ABI 7900HT system using Power Syber® Green assays for targeted gene content and TaqMan assays for copy number references including alpha-satellite sequence and albumin both using a 5′ FAM/3′ BHQ labeled probe (Applied Biosystem). Primer and probe sequences are available upon request. Experiments were carried out using two levels of gDNA inputs for each sample in 384 microwell plates, and in triplicates for each level. Each reaction consisted of a 10 μL system containing 40 ng or 10 ng gDNA input in 5 ng/μL tRNA carrier (Roche Diagnostics), 5 μL Power Syber Green Master Mix® or TaqMan Master Mix, and 0.4 μL primer mixes. Standard curves for references and for target genes using 64, 16, 4, 1, 0.25, and 0.0625 ng of commercial gDNA from a healthy female (Promega) were performed in triplicate or quadruplicate with R square values of 0.99 or above according to a previous method (D'Haene et al., 2010). For positive controls, we did not use any lincRNAs that harbor CNV in our samples as positive or negative controls since this area has not been well explored yet. Therefore, we used known CDKN2A CNV on known CNV-affected samples to test the sensitivity of qPCR. In addition, non-template controls (NTC) were included in triplicates for each plate. Thermal cycling conditions were 95°C for 10 min followed by 40 cycles of 95°C for 15 s and 60°C for 1 min. The unknown sample's DNA quantity is calculated from a linear regression model for a threshold cycle (Ct) relative to DNA starting amount normalized by references. The ratio of DNA quantity means between tumor and normal samples was used to determine somatic copy number changes. Two or more non-call samples were determined in parallel as negative controls. The 2^−ΔΔCt^ method was also used to compare the results from a different analysis.

### Statistical analysis

The proportions of lincRNA, genic and intergenic region-associated CNVs in length were analyzed using the two-tailed Wilcoxon signed rank test. Data distributions are presented as box plots. A Type I Error of 0.05 was used to determine statistical significance. In addition, linear regression was performed between lincRNA and genic regions for all samples.

## Results

### lincRNA CNVs discovery

A total of 14 primary cell lines from 7 matched breast cancers and 7 surrounding healthy normal tissues were investigated by the HumanOmni5 Beadchips. Sample dataset outputs from GenomeStudio were quality-assessed by using log intensity plots between tumor and paired normal samples prior to importation into Nexus. CNV calls were initially generated as discordant logRRs that did not map to the HumanOmni5 reference derived from HapMap samples. These were subsequently improved by using the pair matched analysis where each tumor was adjusted by the corresponding matched normal sample since substantial noise was produced under the unpaired model. lincRNA-associated CNV calls were specifically identified as CNV regions that include or overlap reference lincRNA gene positions. Overall, 86 lincRNA-associated somatic aberration events, including copy gains/losses and allelic imbalances ranging in size from 1.2 Kb to greater than 18 Mb, were predicted among the seven tumor-normal paired samples (**Tables 2** and **S2**). One hundred and twenty-two lincRNAs were affected by CNV genomic regions of over 86 Mb covering from 0.14% to 100% overlapping aberrations; the majority of events were seen in copy number losses (**Tables S2** and **S3**).

**Table 2:** **Large-scale lincRNA involved genomic copy number changes and allelic imbalances detected by Illumina HumanOmni5 array**.

**Case ID**	**CN gains**		**CN losses**		**Allelic imbalances**	
	**Cytoband**	**lincRNAs (n)**	**Cytoband**	**lincRNAs (n)**	**Cytoband**	**lincRNAs (n)**
BC-1					10q11.22	1
					9p12–p11.2	11
BC-2					6p11.2	1
BC-3	9p24.1	1	3q26.32^a^	1	4q13.3	1
			4p15.1	1	10q11.22	1
			7p11.2	1		
			14q32.33	1		
			20p11.1	1		
			10q11.22	1		
BC-4			1q44	3		
			2p25.3	3		
			3p26.3	6		
			4q35.2	3	8p23.2	1
			5p15.33	1	15q11.1– q11.2	8
			8p23.3	1		
			9p24.2	1		
			10p15.3	1		
			11q25	3		
			12p13.33, 12q24.33	5		
			13q34	3		
			15q26.3	1		
			21q22.3	3		
BC-5	11p15.5	1	1q44	1		
	Xq13.1	4	2p25.3	5		
			3p26.2,3p26.3	6		
			4q35.2	7		
			6q27	6		
			7q36.3	2		
			8p23.2	1		
			10p15.3	1		
			12q24.33, 12p13.33	1		
			13q34	3		
			15q26.3	1		
			18p11.31,18p11.32	2		
BC-6	9p21.3–p21.2	1	9p24.1–p21.3^b^, 9p24.3–p24.12	27	2p11.1	1
					9p12–p11.2, 9p21.3–p21.2	12
					10q11.22	3
BC-7					10q11.22	3

a Homozygous Copy Loss.

b LOH.

### CNV resistance of lincRNA genes

**Figure 1:**
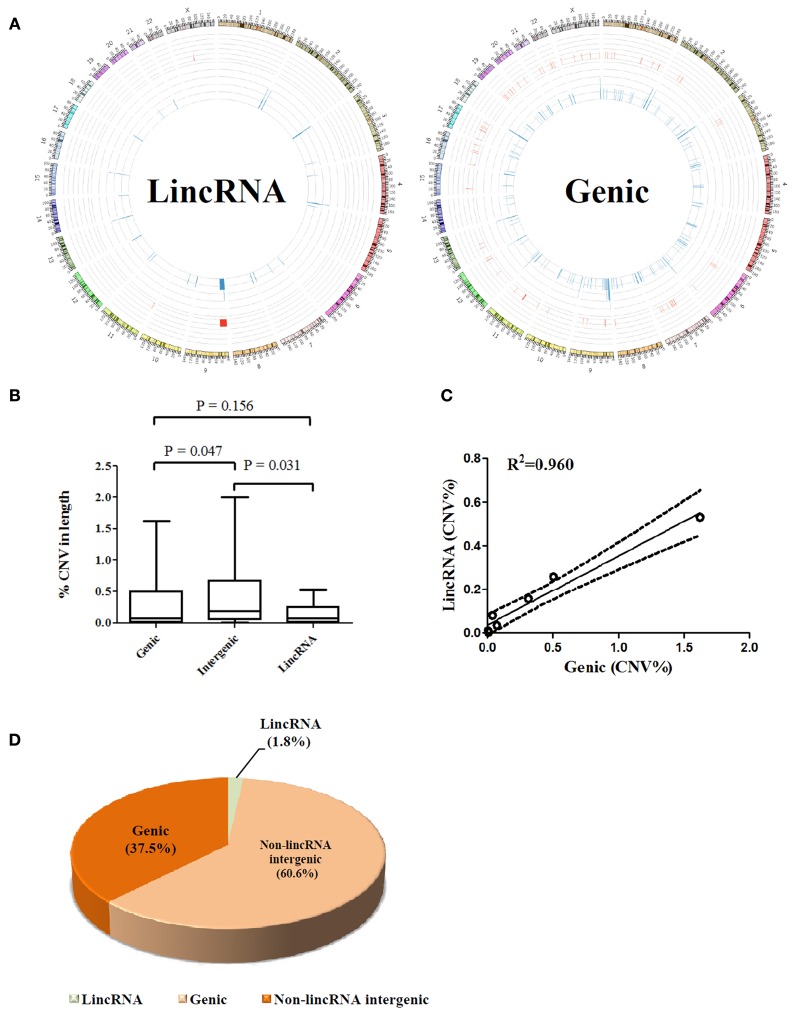
**(A)** Whole genome view of CNV frequencies for lincRNA overlapped (Left) and protein coding gene overlapped (right) regions using an aggregate of seven matched tumor-normal pairs; frequencies were illustrated using Circos software (Krzywinski et al., 2009) in an outward direction. Blue in the inner layer represents copy number losses and red in the middle layer presents copy number gains. Y chromosome was removed from the outer layer. **(B)** Comparison of percentages of CNV affected base pairs in genic, intergenic and lincRNA regions to the total size of each region, respectively; values from seven paired samples were averaged. **(C)** Correlation between percentage of lincRNA overlapped and genic overlapped CNV to the total size of CNV across seven samples. **(D)** Proportion analysis of CNV (non-lincRNA intergenic length = intergenic length–lincRNA length). All calculations were performed on the basis of a female genome.

The overall view for lincRNA gene-associated CNVs and protein-coding gene harbored CNVs is illustrated in Figure 1A. Unlike protein-coding genes where CNVs are generally scattered through each chromosome, lincRNA associated somatic CNVs appear to demonstrate an uneven distribution throughout the genome in the seven tumor-normal pairs. lincRNA associated copy number losses were preferentially clustered to genomic regions at close proximity to the telomere of chromosomes whereas few copy number gains were detected in the central regions.

A further dissection of each CNV-affected region into genic, lincRNA gene, and intergenic (exclude lincRNA genes) associated segments showed only 1.8% of the aberrations fell within the footprint of lincRNAs compared to 37.5% of protein coding genes and 60.6% of the non-lincRNA intergenic segments (Figure 1D). Obviously, affected protein coding genes outnumbered lincRNA associated aberrations over the percentage estimated by chance (40%). Interestingly, although the intergenic region represents a higher proportion of CNVs than the genic region, lincRNA region appears to harbor a lesser proportion of CNVs than the genic region. This is reflected by a 2.4-fold higher mean proportion of CNVs of the genic region compared to that of the lincRNA region (*p* > 0.05, Figure 1B). The finding that lincRNA genes may represent a lesser portion of breast cancer genomic abnormalities may suggest that they are probably less susceptible to genomic aberrations than the protein coding genes in breast cancers. In addition, there is a linear relation (*R*^2^ = 0.96) between lincRNA and genic region (percentage of CNV in length) across 7 samples (Figure 1C).

### Inter-individual heterogeneity in lincRNA CNVs

**Figure 2:**
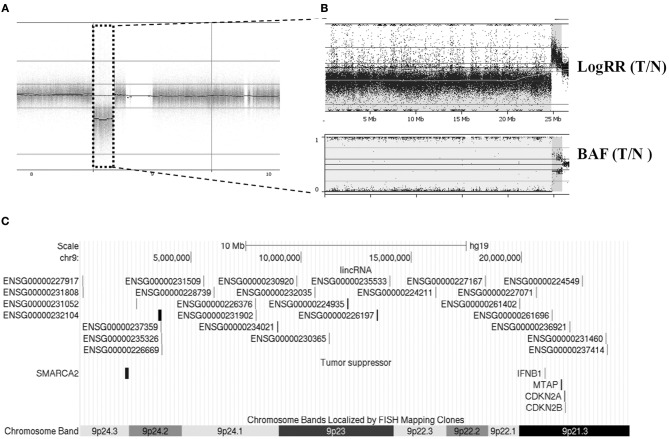
**An example of CNV case analysis revealed large chromosomal CN loss and LOH at chromosome 9 in patient BC-6. (A)** Whole genome view from chromosome 8–10. **(B)** An enlarged LogRR and BAF view of chr9p showed complex aberrations of this region. **(C)** Twenty-seven CNV-affected lincRNA genes and tumor suppressor genes were illustrated across this region.

There is tremendous evidence showing that cancer genomes are complicated and heterogeneous between individuals and even between tumors within an individual. With complicated nature of tumors *per se*, it is not surprising to find the heterogeneity in the somatic alterations in our samples. Some cancers carried many or large regions of changes while some exhibited few or none. For example, Figure 2 depicts data from the primary breast cancer BC-6 showing substantial allelic losses (downward shifts in the adjusted logRRs relative to the baseline, Figure 2B) and one copy number duplication or LOH (BAFs are clustered around values of 0 and 1 corresponding to genotypes AA and BB, respectively, Figure 2B) on chromosome 9p23, which affected over 24 Mb base pairs in length and 27 lincRNA (Figure 2C). By contrast, sample BC-1 most likely remained unchanged except one lincRNA gene harboring allelic imbalances (Table 1). However, a consensus high frequency of allelic imbalances was observed in 10q11.22 in 4 out of 7 tumors, which affected 4 lincRNAs in this region.

### Selective lincRNA CNV validation

**Figure 3:**
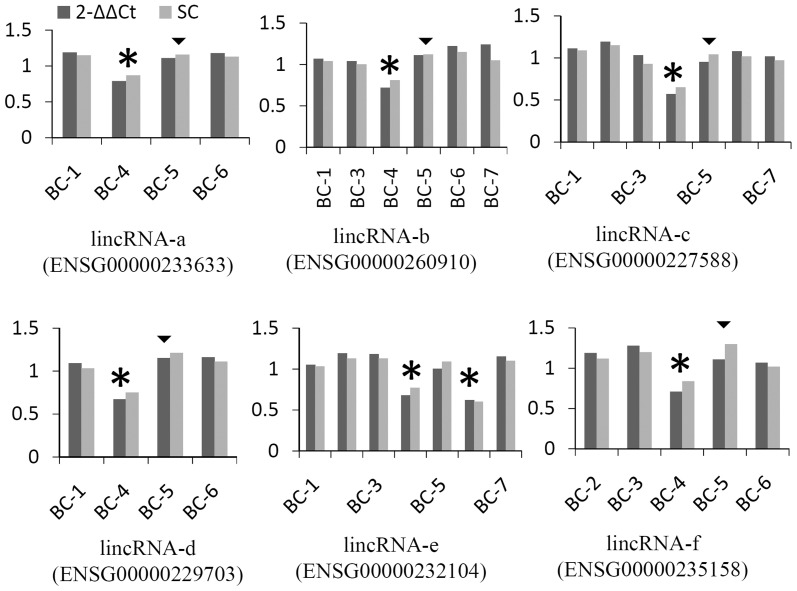
**Examples of validation of lincRNA CNV using qPCR.** Methods of 2^−ΔΔCt^ and standard curve (SC) were compared. Assays from two levels of gDNA input were averaged and values were presented as the ratio of normalized tumor against normal sample using alpha-satellite and albumin as references. Stars represent copy number losses predicted by Nexus and detected by qPCR. Triangles represent copy number losses predicted by Nexus, but not detected by qPCR; a cutoff of 0.2 was used for qPCR (approximately 3 standard deviations of ALB normalized by alpha-satellite).

To further confirm that our identified lincRNAs are expressed in breast tissue even though in the context of cancer, since lincRNAs demonstrated high tissue-specific expression signatures, we queried our lincRNA list against a published expression database (Cabili et al., 2011) and found that all identified lincRNA genes were reported for expression in breast tissues. In addition, qPCR was used to selectively validate lincRNA-associated CNVs showing a predicted recurrence within 7 pairs. Of 33 qPCR reaction assays for 6 lincRNAs, 28 (85%) were in agreement with the prediction from Nexus (Figure 3). All six calls for sample BC-4 were detected while five calls for BC-5 were not detected. Only one recurrent CNV call (lincRNA-f) was detected by qPCR. Aberrations of the six lincRNA-associated regions have been reported by other studies (**Table S4**).

## Discussion

The major objective of this study is to develop a novel approach specifically applied to the identification of breast cancer-associated lincRNAs using high density SNP arrays, by means of which for the first time we report the patterns of lincRNA CNVs in breast cancer genomes. By comparing the differences in genomes between tumors and their matched adjacent normal host counterparts, the resultant copy number aberrations represent bona fide biological gene dosages in tumors, which account for a major portion of cancer genomic structural variations. Although several studies have reported the landscape of the cancer genome by examining hundreds of cancer cell lines (Berger et al., 2011; Curtis et al., 2012; Walker et al., 2012), none of them specifically investigated genomic aberrations associated with lincRNAs. The new HumanOmni5 Beadchip from Illumina consists of about 4.3 million SNPs and provides a comprehensive view of the intergenic portion of the genome, which enables the discovery of small CNVs and defining CNV breakpoints of lincRNAs with a higher informative rate. Since the critical role of certain lincRNA(s) in breast cancers as well as in other cancers has been reported, the development of our approach, implemented in this platform, has been motivated by the need to address the presence of lincRNAs harboring CNVs in breast cancers, and likely contributes to the identification of novel breast cancer-associated lincRNAs.

The challenges raised in the analysis of SNP array data from cancers have been extensively discussed, and results derived from cancer samples may be confounded by numerous factors including (1) the variable tumor purity (potential contamination with normal DNA), (2) the non-uniform nature of cancer genomes including intra-tumor and inter-tumor heterogeneity, (3) aneuploidy or polyploidy features between individuals and chromosomal regions, (4) extensive genomic rearrangements resulting in considerable variations in SNP array data, and (5) technical artifacts produced by SNP array *per se*, which probably requires robust cancer genome specific computational methods (Peiffer et al., 2006; Heinrichs et al., 2010; Yau et al., 2010). Theoretically, these problems are magnified in lincRNAs since their footprints on the genome are less susceptible to CNVs in comparison with protein-coding regions as inferred from Figure 1A. However, in our study, primary breast epithelial cultures enriched tumor purity, which minimized the proportion of normal cell and/or stromal contamination. We also employed the primary cultures at low passage rates (within less than 3 passages) since additional genomic aberrations can be acquired during the course of *in vitro* culture (Stephens et al., 2009). The overview of lincRNA-associated CNVs across all samples visually showed a localized preference to chromosome ends. This pattern may presumably be attributed to the likelihood that chromosomal rearrangements in the breast cancer genomes may physically initiate from the ends of chromosomes. This may partially facilitate interchromosomal rearrangements and intrachromosomal duplications or amplifications, which have been extensively reported in breast cancers as well as in other cancers (Stephens et al., 2009). Moreover, some of the tumor suppressor protein-coding genes present at chromosome ends have been observed in a high degree of deletions (Cao et al., 2011).

For a close examination of individual samples, we found dramatically localized variations among seven primary cancer cell clones from either individual samples (Table 2) or aggregations of all samples (Figure 1) and each harbors its own distinct pattern of genomic CNVs, confirming the heterogeneous nature of tumors. The LOH present in 9p21.3-24.3 in one of the cases may indicate that this region carries coding tumor suppressor genes (e.g., SMARCA2, MTAP, CDKN2A, and CDKN2B, Figure 2C) or even non-coding suppressor candidates, but is less likely a random chromosomal rearrangement event. Future investigations of this region in a larger dataset could be warranted.

It has been shown that the expression signals of intergenic transcripts are generally ~10-fold lower than that of their protein-coding counterpart mRNAs (Cabili et al., 2011). It is, therefore, speculated that gene dosages might be an alternative strategy to compensate for low-expressed lincRNAs, especially in the context of cancer whose hallmark is complex genetic abnormalities. Interestingly, not as we expected, the global pattern of lincRNAs harboring CNVs revealed much more stable genome structures for these RNA than those for protein coding or other intergenic regions, at least in the present breast cancer samples. Since the genomic regions that remain stable in copy number may be essential for maintaining cell survival (Park et al., 2012), lincRNAs could play important roles in cell growth and metabolism to both normal and tumor cells.

In conclusion, we have described a novel approach for genomic copy number and LOH profiling for lincRNA genes in breast cancers using high resolution SNP array. We addressed a number of problems present in SNP-array–based cancer-associated lincRNA CNV analysis. lincRNA genes apparently manifest a relatively more stable manner than coding genes in terms of sensitivity to gene dosage. We identified a couple of lincRNA gene candidates in breast cancers with mostly copy number losses. Further expressional and functional studies on these lincRNAs would be worthwhile for a better understanding of their roles in breast cancer development.

### Conflict of interest statement

The authors declare that the research was conducted in the absence of any commercial or financial relationships that could be construed as a potential conflict of interest.

## Figures and Tables

**Table S1:** **Reference lincRNA list**.

**Table S2:** **Large-scale lincRNA involved genomic copy number changes and allelic imbalances**.

**Table S3:** **Affected lincRNA list**.

**Table S4:** **Query of recurrent lincRNA associated CNVs in the current study against CaSNP database**.
